# Secondary Metabolite Biosynthesis and Regulatory Role of Sucrose in *Bacillus subtilis*

**DOI:** 10.3390/microorganisms14092034

**Published:** 2026-09-12

**Authors:** Minakshi Sinha, Anuradha Tyagi, Shaurya Prakash, Hemlata Kumari, Sumesh Kumar, Vinay Kumar, Antresh Kumar

**Affiliations:** 1 Department of Biochemistry, Central University of Haryana, Mahendergarh 123029, India; 2 College of Medicine, Pennsylvania State University Hershey Medical Centre, Hershey, PA 17033, USAvinayktyagi07@gmail.com (V.K.)

**Keywords:** *Bacillus subtilis*, sucrose, molasses, carbon catabolite repression, quorum sensing, secondary metabolites, surfactin, NRPS, PKS, antimicrobial production, extracellular release

## Abstract

*Bacillus subtilis* is a powerful microbial platform for producing antimicrobial secondary metabolites, yet carbon metabolism, quorum sensing, stress adaptation, and developmental regulation tightly control their biosynthesis. Carbon sources therefore act not only as nutrients but also as regulatory inputs that determine whether the cell prioritizes biomass formation or secondary metabolism. Excess glucose commonly reinforces carbon catabolite repression and suppresses expensive secondary metabolite synthesis pathways. Sucrose and sucrose-rich substrates such as molasses may provide a more balanced carbon flux that supports growth, precursor supply, and transition-phase metabolite production under specific nutritional and physiological conditions. This review discusses how sucrose metabolized through SacP/SacA dependent pathway, fructose- and glucose-derived central carbon pathways, regulate CcpA-mediated CCR, ComQXPA-ComA quorum sensing, Spo0A-AbrB regulation, stress responses, omics-level reprogramming, and efflux through specialized transporters collectively shape antimicrobial metabolite production in *B. subtilis*. Special emphasis is given to surfactin, fengycin/plipastatin, bacilysin, and bacillaene production and release, as they are synthesized using NRPS- and PKS systems influenced by carbon-source-dependent regulation. We propose that sucrose acts as a metabolic-regulatory substrate rather than a simple carbon source in regulating secondary metabolite production. This perspective provides a framework for designing molasses-based, low-cost, and scalable fermentation strategies for enhanced antimicrobial production in *Bacillus* and other microbes.

## 1. Introduction

Microorganisms are an extraordinary source of structurally diverse bioactive compounds, which play significant roles in ecological adaptation, survival, and intermicrobial competition under fluctuating environmental conditions [1]. Among these compounds, microbial secondary metabolites have gained immense scientific and industrial importance due to their tremendous potential for biological activities, such as antimicrobial, antiviral, anticancer, immunosuppressive, and biosurfactant properties [2]. Unlike primary metabolites, which are directly associated with cellular growth and energy generation, secondary metabolites are generally synthesized during the late exponential or stationary phase of growth and are tightly regulated by complex metabolic and environmental signals [3]. In bacteria, the biosynthesis of secondary metabolites is often associated with stress adaptation, nutrient limitation, intercellular communication, and ecological competitiveness [4]. Among Gram-positive bacteria, *B. subtilis* is known as one of the most widely studied model organisms for understanding microbial physiology, metabolic regulation, and extracellular secondary metabolite production. Owing to its Generally Recognised as Safe (GRAS) status, genetic tractability, faster growth, and remarkable secretory molecule capacity, *B. subtilis* has become an important microbial cell factory in pharmaceutical, agricultural, food, and biotechnological industries [5]. It is capable of producing a multitude of secondary metabolites, including lipopeptides, polyketides, bacteriocins, peptides, and surfactants. Important examples include surfactin, fengycin, bacilysin, subtilin, and bacillaene, many of which exhibit potent antimicrobial activities against multidrug-resistant pathogens [6].

The synthesis of secondary metabolites in bacteria largely depends upon cell growth, environmental stress, physiological conditions, and most importantly on culture medium components [7]. Secondary metabolite biosynthesis is primarily governed by polyketide synthase (PKS) gene clusters and large non-ribosomal peptide synthetases (NRPS), which are controlled by local and global regulatory networks [2]. The specific regulatory factor present within or adjacent to the secondary metabolite gene cluster is termed a local regulator. However, global regulators are widely distributed in the genome and influence multiple regulatory systems such as carbon catabolite repression (CCR), quorum sensing pathways, stress-responsive sigma factors, nutrient-sensing mechanisms, and global transcriptional regulators [8]. These systems collectively integrate with different physiological and environmental factors, linked to growth-associated metabolism and biosynthesis of secondary metabolites [9]. Among the various environmental factors affecting secondary metabolism, the nature and availability of carbon sources play a crucial role. Carbon substrates not only provide energy and biosynthetic precursors for metabolite synthesis but also act as signaling molecules that have the potential to modulate gene expression and cellular regulatory pathways [10]. Glucose is preferably used carbon source that is readily metabolized via glycolysis and the pentose phosphate pathway (PPP) to generate energy. However, higher glucose concentrations voluntarily shift the carbon flux to increase cell biomass and primary metabolite synthesis by repressing secondary metabolite biosynthesis through carbon catabolite repression (CCR) activation [11]. Acetyl-CoA is later involved in lipopeptides such as fengycin and surfactin synthesis, and oxaloacetate, α-ketoglutarate, and succinyl-CoA support amino acid-derived secondary metabolite synthesis [12]. NADPH generated from PPP is required for amino acid biosynthesis, fatty acid elongation, redox homeostasis, and NRPS/PKS-mediated assembly of secondary metabolites. Therefore, glucose elicits concentration-dependent physiological responses. Glucose is normally assimilated to support cellular metabolism and meet energy demand, while excess free glucose reinforces CCR to inhibit the synthesis of secondary metabolites [13].

Sucrose is one of the most important sugars utilized during microbial fermentation that can influence the metabolic flux and precursor availability for secondary metabolite biosynthesis. Molasses contains high concentrations of sucrose and minerals, vitamins, trace elements, amino acids, and organic compounds that collectively create a metabolically favorable environment for secondary metabolism [14]. Recent studies demonstrated that sucrose and sucrose-containing substrates, especially sugarcane molasses, not only support secondary metabolite biosynthesis under particular fermentation conditions but also trigger the extracellular release of secondary metabolites in *Bacillus* sp. [15]. The uptake of sucrose in *B. subtilis* is predominantly through the PTS (phosphoenolpyruvate-dependent phosphotransferase system), where sucrose-specific permease, *SacP*, facilitates transport and also phosphorylates sucrose into sucrose-6-phosphate. Subsequently, sucrose-6-phosphate hydrolase eventually convert in to glucose and fructose [16]. Despite its nutritional role, sucrose has also been involved in regulatory signaling events that influence quorum sensing, stress adaptation, and transcriptional regulation of biosynthetic gene clusters [17]. Sucrose-responsive quorum sensing systems termed ComQXPA and the Rap-Phr pathway promote cell-density-dependent activation of surfactin and other antimicrobial biosynthetic clusters in *Bacillus* sp. [18]. In addition, stress-responsive sigma factors, especially σB, contribute to the induction of protective secondary metabolites under osmotic or nutrient-associated stress conditions generated during sucrose-rich fermentation [19]. Such nutrient complexity may alter the cellular metabolic state and influence regulatory processes associated with CCR and stress-responsive pathways, and enhance quorum-sensing-mediated signaling that further favors secondary-metabolite biosynthesis. Furthermore, the osmotic and nutritional characteristics of molasses may influence cellular responses that favor the secretion of extracellular antimicrobial compounds as part of microbial survival and competitive strategies. The relationship between sucrose metabolism and secondary metabolite regulation is increasingly recognized as a multifactorial process involving metabolic flux redistribution, global transcriptional control, energy homeostasis, signaling cascades, and extracellular secretion systems [10]. Despite substantial progress made in understanding secondary metabolism in *B. subtilis*, the mechanistic relationship between sucrose utilization, carbon regulatory pathways, and extracellular release of secondary metabolites remains unexplored. The literature has focused extensively either on fermentation optimization or on isolated regulatory pathways. However, integrated connecting link of sucrose responsive cellular metabolism, signaling cascades, secondary metabolite biosynthetic gene activation, and their extracellular release mechanism is less explored. Therefore, this comprehensive review tends to interconnect different processes essential for current knowledge.

The present review aims to analyze the role of sucrose and sucrose-rich substrates in the regulation of secondary metabolite synthesis and extracellular release in *B. subtilis*. It also focuses on sucrose uptake systems, central carbon metabolism, carbon catabolite regulation, quorum sensing pathways, stress-response mechanisms, activation of NRPS/PKS biosynthetic gene clusters, and extracellular secretion processes involved in antimicrobial metabolite production [9]. Additionally, the review highlights the industrial significance of molasses-based fermentation systems and discusses emerging perspectives in metabolic engineering, systems biology, and sustainable bioprocess development for enhanced microbial secondary metabolite production.

## 2. *Bacillus subtilis*: A Storehouse of Therapeutic Metabolites

**Figure 1:**
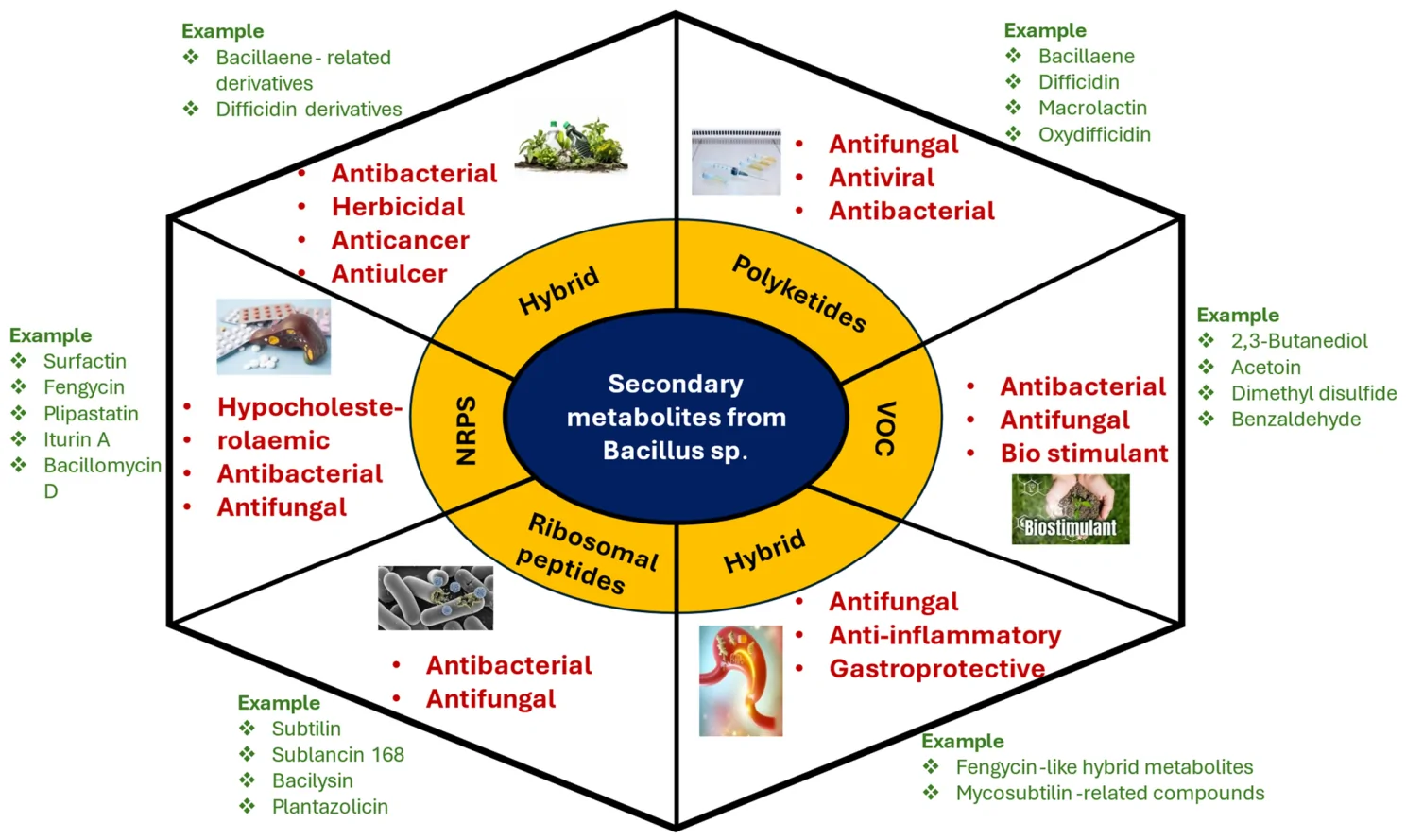
Therapeutic impacts of different secondary metabolites of *Bacillus* sp.

**Figure 2:**
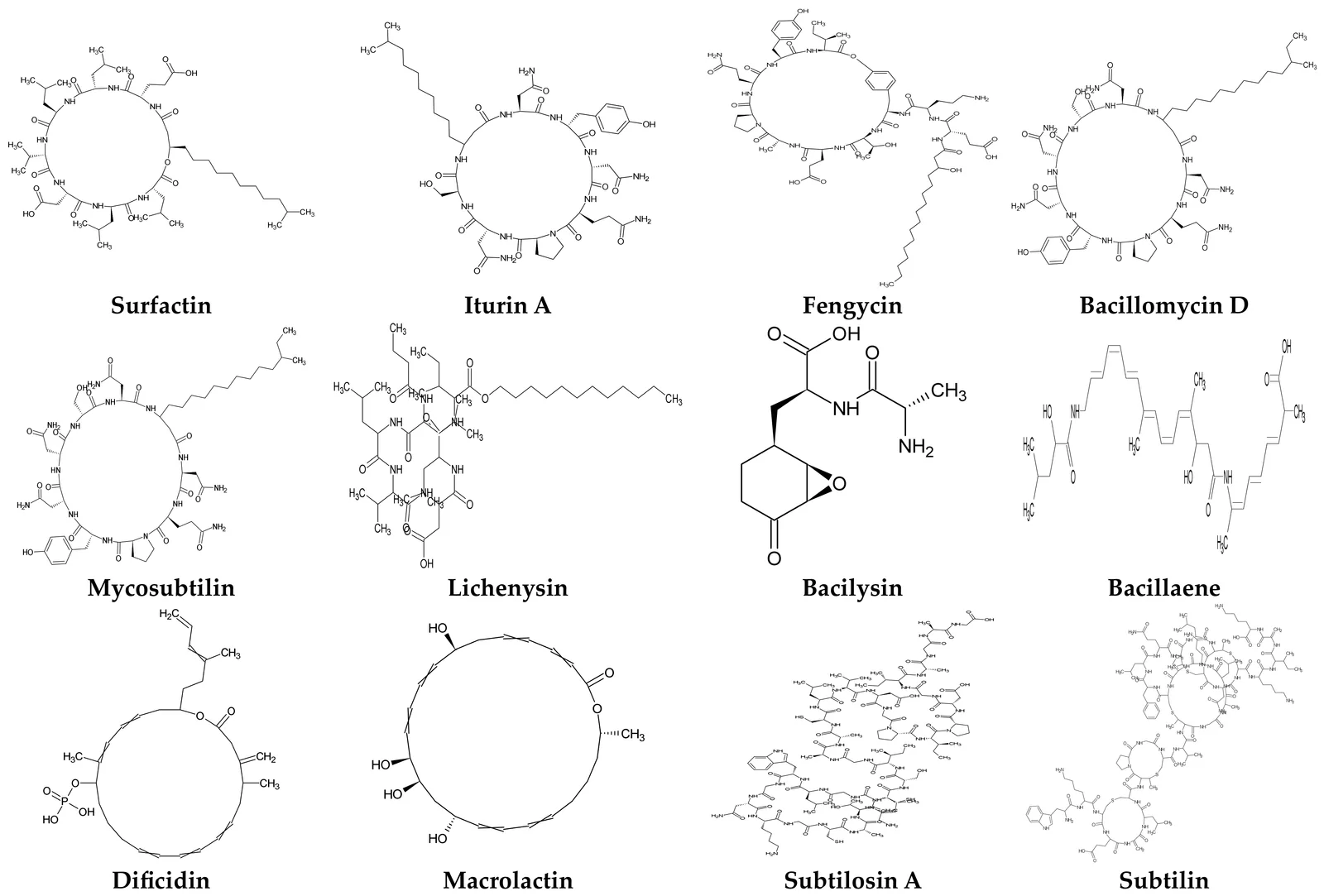

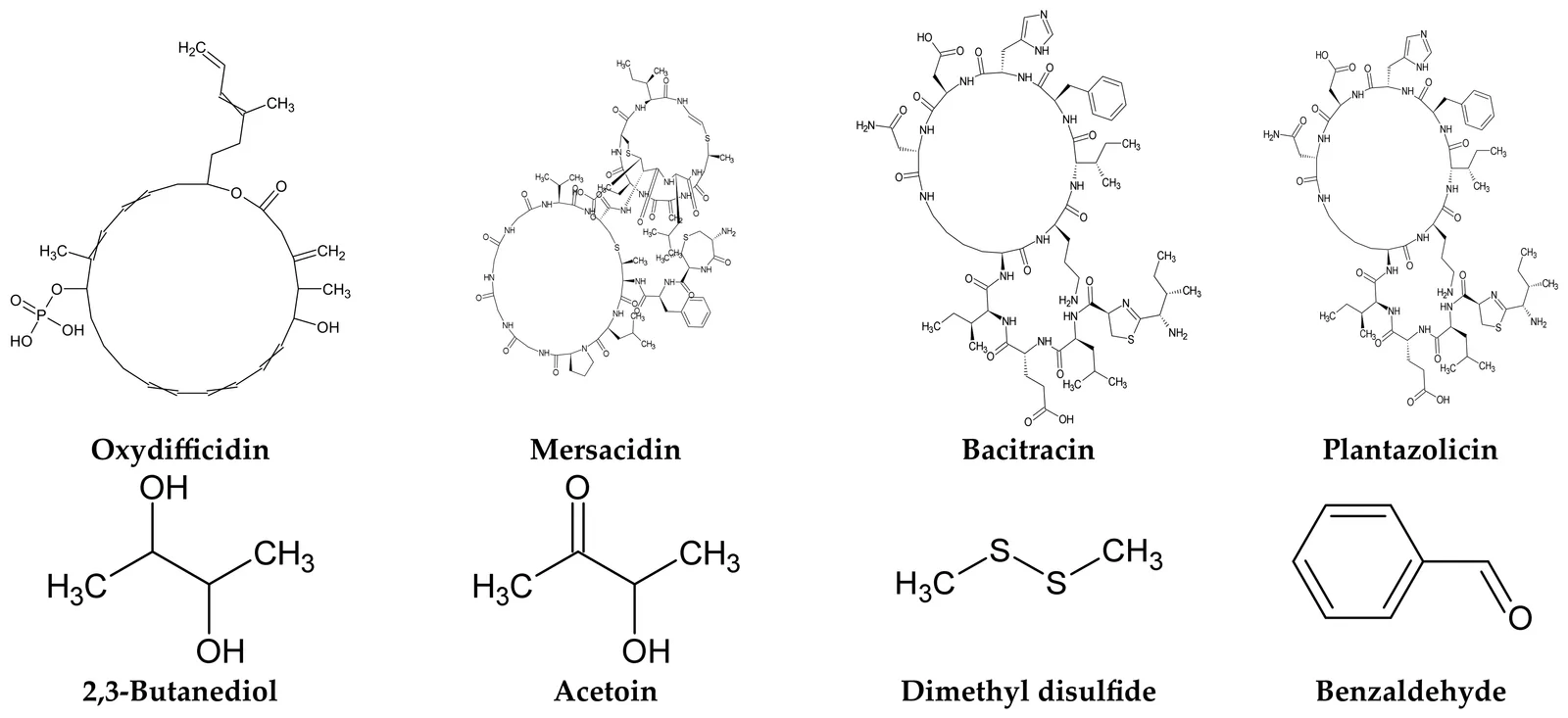
Chemical structure of major therapeutically active secondary metabolites of *Bacillus* sp.

*B. subtilis* is well known for its ability to produce a multitude of structurally diverse extracellular bioactive secondary metabolites with important ecological, pharmaceutical, and industrial applications. These specialized metabolites are synthesized in the late exponential or stationary growth phase, which enables bacterial survival by inhibiting competing microorganisms and facilitates environmental adaptation [6,9,20]. Genomic studies revealed that *B. subtilis* alone contributes up to nearly 4–5% of its genome to secondary metabolite biosynthetic gene clusters, highlighting the remarkable biosynthetic capacity of lipopeptides, nonribosomal peptides, polyketides, and volatile organic compounds (VOCs). The major biosynthetic classes, their representative metabolites, and their associated bioactivities are summarized in Figure 1. The extraordinary diversity of secondary metabolite synthesis in *B. subtilis* is primarily attributed to the involvement of NRPS and PKS systems [9]. Bacillus species synthesize a diverse range of structurally variants with broad pharmaceutical and industrially relevant bioactive secondary metabolites. The structure of some of *Bacillus* sp. derived secondary metabolites are summarized in Figure 2. Unlike ribosomal-mediated peptide synthesis, NRPS pathways operate independently of messenger RNA templates and allow incorporation of unusual amino acids into metabolite structures. Similarly, PKS systems generate complex polyketides through repeated condensation of acyl-CoA units. The modular organization of NRPS and PKS enzymes provides enormous structural flexibility and significantly contributes to metabolite diversity [6].

Lipopeptides-surfactin, fengycin, iturin-like compounds, and others—belong to the peptide synthetase (NRPS) family. Surfactin, a potent biosurfactant encoded by srfA genes in *Bacillus* sp., elicited potent antibacterial, antiviral, antibiofilm, swarming motility, and quorum sensing attributes. It disturbs lipid membrane integrity, leading to cell death [21]. Fengycin is scyclic lipopeptide exhibited a strong antifungal activity against filamentous fungi [22]. Iturin-family lipopeptides tend to cause pore formation in fungal membranes, resulting in leakage of cellular ions and metabolites. Both of them effectively inhibit fungal pathogens such as Rhizoctonia, Fusarium, and Botrytis, reducing dependence on synthetic fungicides [23]. The synergistic effect of fengycin, surfactin, and iturin significantly enhances ecological competitiveness to suppress the growth of neighboring microbial species [23]. *Bacillus* polyketides, such as bacillaene, difficidin, and macrolactin, exhibit broad-spectrum antibacterial properties. These compounds interfere with essential bacterial processes such as protein synthesis, membrane stability, and cellular respiration. A study showed that difficidin shows strong antibacterial activity against both Gram-negative and Gram-positive pathogens, whereas bacillaene inhibits prokaryotic protein biosynthesis. The biosynthesis of these metabolites involves large multifunctional PKS complexes, sequential condensation and modifications of acyl-CoA precursor molecules [24]. The ribosomally synthesized antimicrobial peptides are commonly termed bacteriocins. Bacilysin is one of the most well-characterized peptide antibiotics produced, which is regulated by the *bac* operon in *B. subtilis.* Bacilysin synthesis is strongly influenced by nutrient availability and the quorum sensing pathway [25]. Bacilysin inhibits glucosamine synthetase involved in bacterial cell wall synthesis, thereby suppressing growth of competing microorganisms [26]. *B. subtilis* also produces a different range of volatile organic compounds (VOCs) such as ketones, alcohols, aldehydes, and sulfur-containing molecules that contribute to microbial communication and environmental adaptation. VOCs produced by *Bacillus* sp. stimulate plant growth and induce systemic resistance against phytopathogens without direct physical interaction. Such volatile metabolites therefore play important roles in rhizosphere colonization and sustainable agricultural applications [27]. At nutrient limiting condition cell reprogram the transcriptional machinery required for their survival and competition [28]. This temporal organization is crucial, especially at the exponential stage for metabolite production. Therefore, carbon source selection is a critical growth parameter, considered with the growth phase for secondary metabolite production [29]. The production of secondary metabolites in *Bacillus* sp. is mainly regulated and controlled by the growth phase, nutrients present, environmental stress, etc. Table 1 represents different secondary metabolites produced by various strains, their biological activities, and regulatory factors influencing them.

## 3. Role of Sucrose in Secondary Metabolite Synthesis

The microbial growth and bioactive secondary metabolite biosynthesis are primarily influenced by the availability of different carbon sources, including glucose, fructose, sorbitol, glycerol, sucrose, mannitol, and others. Among these, glucose is preferentially utilized and supports biomass production and primary metabolism. However, the presence of excess glucose in media suppresses secondary metabolite synthesis via activation of carbon catabolite repression (CCR) and suppresses the biosynthesis of high-energy-demanding secondary metabolites. Consequently, due to low cost, broad availability, and high metabolite yield, sucrose and sucrose-rich substrates have gained attention among the various carbon substrates in industrial fermentation. These substrates have high potential to support metabolite production under optimized nutrient conditions [49]. Sucrose balances cellular energy and metabolic flux required for cell metabolism [16].

**Figure 3:**
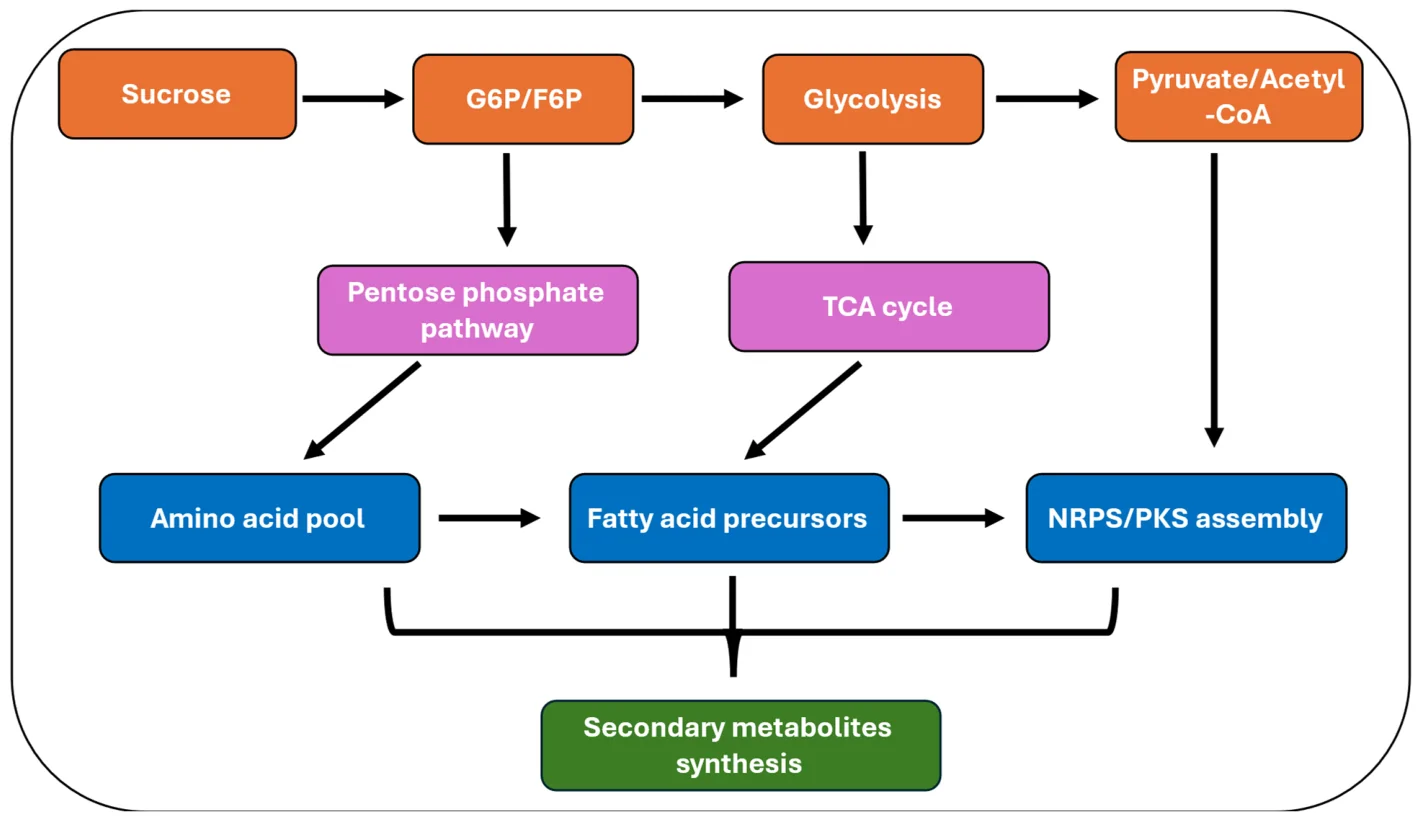
Sucrose-derived metabolic fate supports secondary metabolite synthesis.

The metabolic fate of the sucrose-derived carbon source and its contribution toward precursor generation, energy production, and NRPS/PKS-mediated secondary metabolite assembly are illustrated in Figure 3. Various factors such as pH, incubation period, nitrogen, and dissolved oxygen tend to regulate the uptake and assimilation of sucrose to fulfil energy-demanding secondary metabolite biosynthetic processes [10]. Secondary metabolite production depends on interacting regulatory mechanisms and on how the sucrose-derived carbon is distributed between biomass formation, energy generation, and precursor synthesis.

Sucrose metabolism influences the gradual release of carbon sources from growth media by redirecting carbon flux as compared to glucose metabolism, resulting in balanced metabolic activity, potentially reducing carbon catabolite repression (CCR) associated pathways. This metabolic environment favors activation of biosynthetic gene clusters and prolonged stationary-phase metabolism, and increased secondary metabolite biosynthesis [50]. However, sucrose-mediated activation of secondary metabolites is not universal and remains strain-specific.

Studies from *Bacillus* strains revealed that a sucrose-rich medium boosts secondary metabolite production in *Bacillus* sp. under specialized conditions. For instance, the combined effects of carbon flux from sucrose and other contributory factors increased biosynthesis of surfactin in *B. subtilis* UFPEDA 438 [16]. Sucrose with other media components (soybean meal and Mg^2+^) escalated biosynthesis of iturinA in *B. subtilis* BH072 [51]. These findings collectively support that sucrose supplemented with other components favors an increase in secondary metabolite biosynthesis in *B. subtilis*.

Sugarcane molasses, a rich source of sucrose, has also been used as a carbon source for secondary metabolite synthesis in various *Bacillus* sp., and its magnitude of response varies with strains and experimental conditions. For instance, *B. subtilis* ANR88 was grown with different agro-industrial substrates: molasses, orange peel, potato peel, whey, and bagasse, for lipopeptide production. Results showed that molasses increased lipopeptide production the most, with a yield of 0.24 g/L [52]. Similarly, molasses also positively regulates surfactin synthesis in *B. subtilis* BS5 [53]. Molasses has also been supplemented as a carbon source for large-scale synthesis of lipases, proteases, and carbohydrate-active enzymes in the food, chemical, pharmaceutical, and textile industries for improvement of yield [54,55]. Molasses supplemented with dextrose and casein significantly escalated the antifungal metabolite echinocandin B synthesis in *Aspergillus nidulans* [4]. This distinction is particularly important because molasses is chemically more complex than other purified sugars. A recent proteomics study further supports the role of fructose-responsive lipopeptide production in different *Bacillus* sp. In *B. amyloliquefaciens* fmb-60 supplemented with fructose, NRPS-mediated fengycin production was enhanced by increasing amino acid metabolism, fatty acid biosynthesis, energy generation, and stress response, resulting in redistribution of fengycin biosynthesis [56]. Comparative evidence from non-*Bacillus* strains also demonstrated that sucrose significantly affects secondary metabolite biosynthesis. For example, *Streptomyces* sp. KGG32 also produced higher levels of antimicrobial agents when sucrose was used as the sole carbon source under aerobic fermentation conditions, with increased zones of inhibition against *S. aureus* and *E. coli*, respectively [57]. Glucose, glycerol, starch, and maltose exhibited lower antimicrobial activity than sucrose, indicating that sucrose is not merely a growth-supporting carbon source but is closely associated with enhanced production of antimicrobial metabolites. Similarly, *Streptomyces tritolerans* DAS 165T also increased antimicrobial secondary metabolites [58]. In contrast, *Streptomyces* sp. 201 showed maximum growth and antimicrobial metabolite production with mannitol, followed by sucrose and glycerol, suggesting that sucrose was not the optimum carbon source, but it supports significantly in bioactive metabolite production in certain Streptomyces strains [59]. The effects of sucrose on secondary metabolite synthesis have also been reported in fungi. Mathan et al. (2013) revealed that sucrose-supplemented media encouraged biomass and antimicrobial secondary metabolite production in *Aspergillus terreus* KC 582297, as compared to all tested carbon sources [60]. This observation further supports the view that sucrose can serve as an efficient carbon source for antibiotic or bioactive metabolite production in both bacteria and fungi under optimized growth conditions and strain-specific. However, it has also been reported that some organisms were not capable of utilizing sucrose at industrial-scale fermentation. The sucrose utilization pathway was explored by overexpressing a sucrose-utilizing gene in the bacterial expression system. For instance, a recombinant *E. coli* XL1-Blue strain overexpressing β-fructofuranosidase was capable of utilizing sucrose as the sole carbon source, and synthesized 3.76 g/L poly(3-hydroxybutyrate) and 1.82 g/L poly(3-hydroxybutyrate-co-lactate) [61]. Similarly, co-expression of sucrose permease, invertase, and fructokinase enzymes promoted effective transport and metabolism of sucrose, leading to increased D-glucaric acid production with a yield of 0.142 g/g sucrose [62]. With advances in metabolic engineering, sucrose is increasingly used as an alternative low-cost substrate for the microbial production of high-value biomolecules, including carotenoids, functional sugars, and amino acids. A genetically engineered *E. coli* strain expressing sucrose phosphorylase along with UDP-sugar biosynthetic enzymes escalated quercetin 3-O-galactoside and quercetin 3-O-rhamnoside synthesis. Similarly, other recombinant strains were also capable of utilizing sucrose-derived UDP-glucose for astragalin production. Recombinant *Clostridium tyrobutyricum* and *Pseudomonas putida* induced butanol and rare sweetener 5-ketofructose synthesis, respectively, in the presence of sucrose as a carbon source [63]. Similar to sucrose, sugarcane molasses is also traditionally regarded as a low-cost carbon feedstock used almost 55 million tons per annum in industrial fermentation [45].

The findings from other *Bacillus* species and microorganisms provide comparative insight into possible carbon-regulatory mechanisms, but do not constitute direct evidence for their operation in *B. subtilis.* This distinction of secondary metabolite production in *Bacillus* and non-*Bacillus* sp. provides an important framework for evaluating the potential regulatory role of sucrose in secondary-metabolite biosynthesis and for identifying mechanisms that remain to be experimentally established. The empirical effects of different carbon sources on secondary metabolite production in *Bacillus* sp. are summarized in Table 2.

## 4. Sucrose-Mediated Regulation of Secondary Metabolite Synthesis

### 4.1. CCR-Mediated Metabolic Response

Carbon catabolite repression (CCR) plays a central role in carbon-source preference and secondary metabolism. It shifts the cellular response toward biomass production rather than secondary metabolite synthesis [10]. In response to glucose utilization level of ATP and fructose-1,6-bisphosphate (F1,6-BP) elevated, leading to HPr phosphorylation by the kinase/phosphorylase at serine-46 that interacts with the transcriptional regulator CcpA to make a complex CcpA-HPr-Ser46-P. This complex recognizes the CRE (catabolite-responsive elements) located in the promoter region of specific target genes, represses genes needed for using other carbon sources, and also indirectly suppresses secondary metabolite biosynthesis. Under glucose-rich conditions, *B. subtills* preferentially allocates metabolic resources to cell growth, protein synthesis, and cell division rather than high-ATP-requiring secondary metabolite synthesis [10]. The extent of CCR depends on the carbon source, demonstrating that sucrose exerts substantially weaker CcpA-mediated CCR (*xynPB* reporter) than glucose in *B. subtilis* [68]. Thus, sucrose utilization may impose a comparatively weaker CCR response than glucose, which provides a less restrictive carbon-regulatory environment and favours secondary metabolite biosynthesis under appropriate conditions in *B. subtilis*. Further, the relevance of weaker CCR to secondary metabolite production is supported by comparative evidence from *B. amyloliquefaciens*. This study states that when glucose is depleted, CCR is relieved and surfactin production was associated with the subsequent utilization of alternative carbon sources [69]. Sucrose utilization occurs primarily by the conserved phosphoenolpyruvate (PEP) dependent phosphotransferase system (PTS) in bacteria. SacP, a sucrose-specific transporter, uptakes extracellular sucrose into the cytoplasm. PTS also acts as a nutrient-sensing system, which reflects carbon availability and influences global regulation [17]. During transport, phosphoenolpyruvate (PEP) donates a phosphate group through a cascade involving Enzyme I and HPr proteins, resulting in the formation of sucrose-6-phosphate during membrane translocation. Later, sucrose-6-phosphate is hydrolyzed by SacA, a sucrose-6-phosphate (S6P) hydrolase, to generate glucose-6-phosphate (G6P) and fructose, which initiates glucose- and fructose-derived branches. Although sucrose metabolism provides metabolic intermediates of different metabolic pathways, its influence on secondary metabolite production cannot be explained only by carbon supply.

The secondary metabolite biosynthetic response finally depends on how sucrose-derived carbon (mainly fructose) gets integrated with cellular regulatory networks that control the shift from active growth phase to biosynthetic phase. Cell glucose directly enters glycolysis and the PPP for metabolism. However, fructose needs to be phosphorylated before entering the central carbon metabolism. Fructose is phosphorylated through the fructose-specific phosphotransferase system or ATP-dependent fructokinase to produce fructose-1-phosphate (F1P) or fructose-6-phosphate (F6P). F1P is then converted into fructose-1,6-bisphosphate (F1,6-BP) by the enzyme 1-phosphofructokinase and enters the glycolysis pathway and downstream into the TCA cycle for ATP generation, amino acid precursor biosynthesis, carbon flux, and biomass formation [70]. Earlier studies revealed that fructose-containing media enhanced polyketides, pigments, exopolysaccharides, biosurfactants, and antibiotic production in microbes [71].

At the transition to the stationary phase, sucrose-derived carbon flux may interact with global regulatory mechanisms such as quorum sensing, ComA-dependent activation of the surfactin biosynthetic operon, Spo0A-linked developmental regulation, and precursor availability to regulate bioactive metabolite biosynthesis [31]. This indicates that the effect of sucrose on secondary metabolite production is not simply nutritional, but depends on a coordinated metabolic-regulatory response involving carbon flux, growth phase, and global regulation [10]. Thus, sucrose and sucrose-rich sources act as metabolic-regulatory substrates rather than simple carbon sources.

### 4.2. Quorum Sensor-Mediated Response

The synthesis of secondary metabolites in *B. subtilis* is also regulated by a density-dependent cellular communication system collectively termed quorum sensing (QS). Quorum sensing coordinates with gene expression in response to altered cell density through secretion and detection of extracellular signaling molecules. This regulatory mechanism allows bacterial communities to synchronize biofilm formation, competence development, sporulation, stress adaptation, and bioactive metabolite production. In *B. subtilis*, QS serves as a critical regulatory link between environmental sensing and activation of biosynthetic pathways responsible for antimicrobial compound production [72].

Unlike Gram-negative bacteria that primarily utilize acyl-homoserine lactones as signaling molecules, Gram-positive bacteria employ small-secreted peptides that act as autoinducers. The signaling network of *B. subtilis* comprises several interconnected systems, including the ComQXPA-ComA and Rap-Phr pathways. ComQXPA-ComA has a well-established role in surfactin regulation, whereas Rap-Phr systems modulate broader regulatory networks that influence secondary metabolism. These pathways collectively integrate population density signals with global transcriptional regulators that regulate gene expression of biosynthetic gene clusters [73]. In the ComQXPA signaling system, the ComX peptide is modified by ComQ to produce its mature, active pheromone form. As the bacterial population grows, ComX gradually accumulates outside the cells. Once it reaches a sufficient concentration, it is detected by the membrane-bound histidine kinase ComP. This interaction activates ComP through autophosphorylation, after which the phosphate group is transferred to the response regulator ComA. The activated ComA protein then acts as a transcriptional regulator, switching on genes involved in competence development and the production of secondary metabolites [45]. One of the most important targets of phosphorylated ComA is the srfA operon, which encodes the NRPS complex responsible for surfactin biosynthesis. Previous studies demonstrated that ComA is essential for the activation of srfA transcription, which tends to establish a direct molecular connection between antimicrobial metabolite production and quorum sensing [30]. Importantly, increased expression of quorum-sensing-associated genes does not necessarily indicate increased signaling activity. Transcription-level changes alone do not establish increased signaling activity or extracellular metabolite transport. Thus, the possible connection between sucrose availability and quorum-sensing-mediated secondary-metabolite regulation should be considered condition-dependent unless supported by appropriate functional or genetic evidence. As cell number increases, enhanced ComA activity results in elevated surfactin synthesis, enabling bacterial populations to coordinate production of this energetically expensive metabolite only when sufficient cellular resources and population size are available [30]. Surfactin itself plays a dual role within the bacterial community. In addition to its antimicrobial and biosurfactant activities, surfactin functions as a signaling molecule that can trigger biofilm formation, multicellular behavior, and cell differentiation. Surfactin also stimulates potassium leakage from cell membranes, activating signaling cascades for the development of biofilm and community organization [74]. This finding highlights the intricate relationship between secondary metabolites and quorum sensing networks in *B. subtilis* [75]. Rap-Phr signaling system is another regulatory mechanism influencing quorum sensing-mediated metabolite production. Members of the Rap family function as phosphatases that negatively regulate response regulators such as ComA and Spo0F. Their activity is controlled by secreted Phr peptides, which accumulate extracellularly in a population-density-dependent manner. Following re-import into the cell through oligopeptide transport systems, Phr peptides inhibit Rap proteins and relieve their suppressive effects on downstream regulators. Through this mechanism, the Rap-Phr network fine-tunes quorum-sensing responses that influence downstream developmental and secondary-metabolite-associated processes under specific physiological conditions [31]. The integration of ComQXPA and Rap-Phr systems establishes a highly dynamic regulatory network for the regulation of secondary metabolism, population growth, and environmental adaptations. Several studies have demonstrated that disruption of quorum-sensing pathways significantly reduces the production of surfactin and other antimicrobial secondary metabolites. For instance, a mutation in ComA resulted in disruption of quorum sensing, decreasing the srfA level and surfactin synthesis [76].

Quorum sensing in bacteria has also been explored in stage- specific growth regulation via global regulatory networks. Spo0A, DegU, AbrB, and CodY are reported to be the key regulators involved in interactions of the quorum sensing pathway to coordinate developmental processes and secondary metabolite biosynthesis. Spo0A, regarded as the master regulator of sporulation, indirectly promotes antimicrobial secondary metabolite production by repressing AbrB, a transition-state regulator that restricts the expression of numerous stationary-phase genes. Consequently, activation of Spo0A facilitates the biosynthetic operons involved in antimicrobial compound synthesis [22,77]. Bacilysin, a dipeptide antibiotic synthesized through the bacABCDE/bacABCDEF cluster together with bacG/ywfG in *B. subtilis*. Bacilysin synthesis is induced by the quorum-sensing-associated and global regulatory systems at the transition to the stationary phase [11,26]. Similarly, the polyketide bacillaene, produced by the pksX/pksA-pksS, contributes to microbial competition by inhibiting prokaryotic protein synthesis [6,8]. The production of fengycin, subtilin, and several other bacteriocins is directly or indirectly controlled by quorum-sensing-associated regulators. The coordinated expression of these compounds enables *B. subtilis* to effectively compete with neighboring microorganisms within densely populated ecological niches such as soil, plant rhizospheres, and biofilm communities [6]. Similarly, the quorum sensing module ComQXPA-P srfA dynamically regulates plipastatin biosynthesis in *B. amyloliquefaciens* with a yield of 385 mg/mL [64].

The function of quorum sensing in secondary metabolite production has also been studied in different non-*Bacillus* bacteria. For instance, AHL- and quinolone-based QS systems in *Pseudomonas* species control the synthesis of phenazine, pyocyanin, and pyrrolnitrin bioactive compounds. AHL- and quinolone-based QS systems also control the synthesis of secondary metabolites, population density, biofilm development, and ecological competition [78,79,80]. Similarly, γ-butyrolactone signaling in *Streptomyces* (actinomycete) also activates antibiotic biosynthesis and growth differentiation [81], and *Serratia marcescens* regulates the production of Prodigiosin and carbapenem through AHL-QS along with nutrient composition [82]. The CviI/CviR AHL-based quorum-sensing mechanism in *Chromobacterium violaceum* controls violacein production regulated by cell density [83]. Activation of quorum sensing pathways alters carbon flux distribution, amino acid metabolism, stress response systems, and energy homeostasis, thereby creating favorable conditions for secondary metabolite production [84].

Sucrose- and molasses-mediated secondary metabolite production in *B. subtilis* involves multiple layers of regulation, including sucrose uptake, carbon catabolite repression, quorum sensing, transition-state regulation, and biosynthetic gene-cluster activation-associated pathways. The major genes involved in sucrose utilization, carbon catabolite repression, quorum sensing, transition-state regulation, and antimicrobial biosynthesis are summarized in Table 3. Understanding quorum sensing-mediated regulation is therefore critical not only for elucidating the bioactive role of secondary metabolites but also for developing efficient biotechnological processes aimed at maximizing production of commercially valuable antimicrobial compounds [84,85].

### 4.3. Stress-Responsive

Secondary metabolites are primarily synthesized at the late exponential or stationary phase. This timing reflects the role of secondary metabolites in survival and competition rather than direct biomass generation. Different environmental and physiological stresses such as nutrient scarcity, temperature, idiotrophic growth transition, and microbial competitions influence the production of antimicrobial metabolites in bacteria [96]. The sucrose- or molasses-containing media, under moderate osmotic or nutritional stress, may act as a stimulatory signal for secondary metabolism in *B. subtilis* by promoting transition-state regulation and stress-adaptive responses [97]. Moreover, osmotic and oxidative stress, combined with membrane perturbations, activate the sigma factors that redirect RNA polymerase toward stress-responsive genes. Among these regulators, σB serves as the principal mediator of the general stress response in *B. subtilis*. Activation of σB promotes cellular adaptation to adverse conditions and is associated with increased expression of genes involved in antimicrobial production, biofilm development, and environmental persistence [98]. A bunch of earlier studies have targeted the stress-responsive metabolite production. For example, low-temperature stress in *B. subtills* ATCC6633 escalated Mycosubtillin up to 30-fold [36]. The ScoC, AbrB, and CodY regulators negatively regulate bacilysin biosynthesis in *B. subtilis*, which increased the production of bacilysin under limiting nutrient conditions or idiotrophic transition phase [38]. In another study, Vargas-Bautista et al. demonstrated that bacillaene-associated pks genes in *Bacillus subtilis* are regulated in response to bacterial competition. Their study showed that Spo0A, mainly through repression of AbrB, is important for pks expression, while CodY, DegU, ComA and ScoC further modulate its regulation. This indicates that bacillaene production is controlled by stress-, nutrient-, and competition-responsive regulatory networks rather than being constitutively expressed [39].

Several metabolites also contribute to plant growth promotion by suppressing phytopathogens, stimulating root development, and inducing systemic resistance responses in host plants [6]. According to Bais et al., *B. subtilis* 6051 protects Arabidopsis roots against *Pseudomonas syringae* infection by forming a root-associated biofilm and by producing more surfactin into the environment. This study supports the ecological role of surfactant as a plant-associated defense metabolite that contributes to pathogen suppression and microbial competition survival [99]. Another non-*Bacillus* study also proves the role of nutrient stress in antibiotic production. In *Streptomyces coelicolor* amino acid limitation in nutrient media increases ppGpp accumulation, which further links nutritional stress with morphological differentiation and increases the production of antibiotics such as actinorhodin and undecyl prodigiosin [100,101].

Based on these studies, we can say that beyond direct antimicrobial activity, the secondary metabolites produced by *B. subtilis* perform important ecological functions such as biofilm formation, surface colonization, microbial competition, and rhizosphere establishment to survive stress conditions. Polyketides and peptide antibiotics restrict growth of neighboring microorganisms through diverse actions, including membrane disruption, inhibition of protein synthesis, and interference with cell wall biosynthesis.

**Figure 4:**
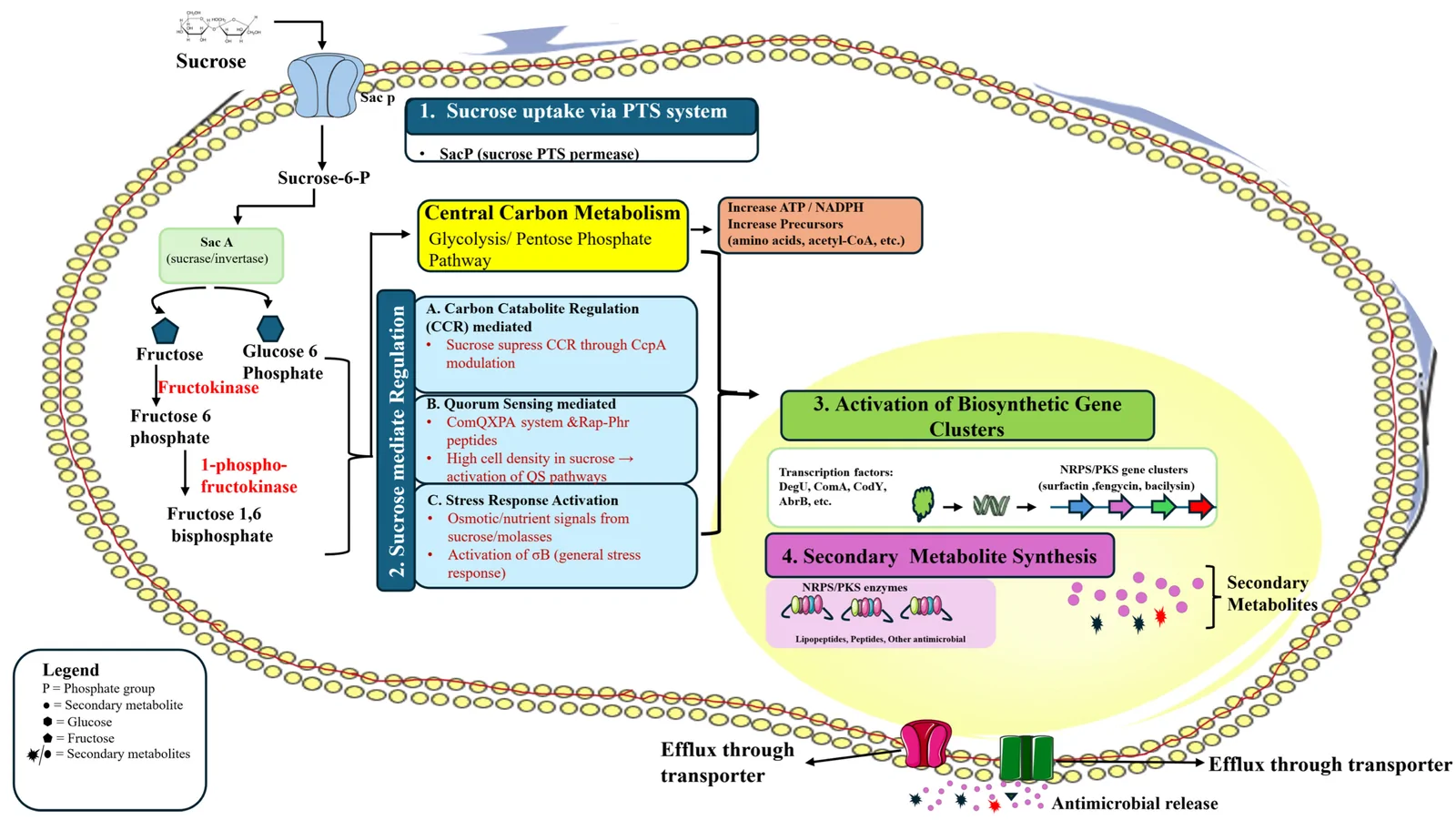
The proposed model represents the potential influence of sucrose availability on secondary metabolite synthesis and their extracellular release.

Understanding these interconnected regulatory mechanisms provides valuable opportunities for metabolic engineering and fermentation optimization aimed at escalating production of antimicrobial metabolites at industrial scale [102]. These dynamic interactions ensure efficient adaptation to altered environmental conditions while maximizing the ecological and biotechnological potential of microbial secondary metabolism [103]. Sucrose- or Molasses-containing media cause a mild osmotic or nutritional stress depends on the concentration and composition. Mild stress can sometimes stimulate secondary metabolism as part of adaptive competition, whereas severe stress conditions are not favored and restrict growth and biosynthetic capacity. This creates a universal pattern that moderate stress signals may be beneficial, but excessive substrate concentration or osmolarity becomes inhibitory [104]. Based on the available evidence, sucrose-mediated enhancement of secondary metabolite production can be considered a multi-layered regulatory process involving carbon uptake, CCR modulation, quorum sensing activation, biosynthetic gene expression, and extracellular transport. A proposed model illustrating potential links between sucrose metabolism, regulatory networks, secondary-metabolite biosynthesis, and extracellular transport in *B. subtilis* Figure 4.

## 5. Role of Sucrose in Extracellular Transport of Secondary Metabolites

The therapeutic potential of microbial secondary metabolite biosynthesis not only depends on their intracellular synthesis, but also on their extracellular release. Different types of transport systems govern the extracellular release of microbial secondary metabolites. The Sec and Tat are two primary pathways for transporting secretory proteins across the membrane. The Sec pathway represents the major secretion route in *B. subtilis*. The N-terminal signal peptides translocate the secretory proteins across the membrane in an unfolded state through the Sec machinery. In contrast, the Tat pathway facilitates the transport of twin-arginine signal peptides containing folded proteins across the membrane. Structurally diverse molecules such as ribosomally synthesized peptides, bacteriocins, lantibiotics, and non-ribosomal lipopeptides are primarily exported through ABC (ATP-binding cassette), MFS (Major facilitator superfamily), and RND (Resistance-nodulation-division) efflux pumps [20].

Sucrose-containing media may influence the biosynthesis and extracellular transporter-mediated efflux of specific secondary metabolites in *Bacillus* sp. by coordinating with carbon uptake, biosynthetic pathways, and transporter-associated self-resistance mechanisms [20]. The extracellular accumulation of antimicrobial metabolites depends on both metabolite biosynthesis and their subsequent export from the cell. However, the relative contribution of biosynthesis and export may vary among metabolites, strains, and cultivation conditions. Transcriptomic analysis revealed that *B. amyloliquefaciens* MT45 cultured in sucrose supplemented media, showed increased expression of *srfA* and *swrC* genes, associated with surfactin production and export. Moreover, expression of global regulators such as ccpA, codY, degU, and spo0A also increased under similar growth conditions. These studies suggest that sucrose supplementation of the growth medium favors the metabolic flux and transport of secondary metabolites [105]. Several molasses-based fermentations also provide an effective low-cost substrate for extracellular biosurfactant and lipopeptide production in *B. subtilis* UFPEDA 438 [15]. In another study on *B. subtilis* YPS-32, molasses was identified as the most suitable carbon source among different tested substrates for inducing production and transport of surfactin [57]. The ribosomally synthesized antimicrobial peptides and bacteriocins primarily require a specific export system for extracellular release. For instance, the subtilin (lantibiotic) in *B. subtilis* ATCC6633 is encoded by a spa gene cluster *spaBTCSIFEGRK*, responsible for synthesis, maturation, export, and regulation of the lantibiotic [45]. Similarly, SunT, an ABC transporter involved in the processing and transport of sublancin in *B. subtilis* [8]. The RND-family transporter YerP/SwrC has also been identified for surfactin efflux [106]. In another study, YcxA and KrsE, proton motive force-dependent transporters linked to inducing surfactin secretion [107]. Moreover, overexpression of YerP, YcxA, YfiS, and KrsE contributes strongly to surfactin export [108]. Although several transporters linked to secondary metabolite export have been experimentally discussed, direct evidence that sucrose or molasses enhance their activity remains limited. Induced transporter gene expression may or may not be included in the increment of extracellular metabolite export. YoeA, a MATE-family transporter reported to improve plipastatin production in *B. subtilis*, and its overexpression increased extracellular transport [107,109]. The transporters for iturin, bacillomycin and mycosubtilin are less clearly defined, though their extracellular accumulation is also likely to require transporter-assisted release and self-resistance mechanisms because these compounds interact strongly with microbial membranes. For other antimicrobial metabolites, such as bacilysin, bacillaene, and difficidin-like compounds reported to efflux through multidrug transporters, ABC exporters, MFS-type pumps, or cluster-associated resistance proteins [110]. The glycopeptide antibiotics, which are not typically products of *B. subtilis*, but glycopeptide-producing actinomycetes provide a useful comparative model. In these organisms, antibiotic biosynthetic clusters often contain dedicated ABC transporters that export the product and protect the producing cell [111]. *Streptomyces albus* BK3-25 showed that high salinomycin production was associated with increased expression of several transporter genes, indicating extracellular metabolite transport and resistance [112].

## 6. Future Perspectives and Conclusions

Despite substantial progress in understanding secondary metabolism in *B. subtilis*, several challenges remain constant regarding optimization of metabolite production and regulatory control. Future studies should integrate genomics, transcriptomics, proteomics, metabolomics, and fluxomics to understand how sucrose and molasses regulate secondary metabolite production in *Bacillus* sp. Genomics can identify biosynthetic gene clusters, transcriptomics can reveal carbon-source-dependent activation of regulatory and biosynthetic genes, proteomics can confirm the abundance of NRPS/PKS enzymes and precursor-supply proteins, metabolomics can quantify antimicrobial products, and fluxomics can determine how carbon is redistributed toward acetyl-CoA, amino acids, NADPH, and fatty-acid precursors. Such integrated omics approaches will be essential for rational optimization of sucrose- and molasses-based fermentation systems. This is mandatory to unravel the complex interactions among carbon metabolism, quorum sensing, stress responses, and biosynthetic gene regulation.

Despite these advances, several important mechanistic questions remain unresolved. Future studies should elucidate the molecular basis of strain-dependent sucrose responses, distinguishing the contribution of carbon flux redistribution from signaling and regulatory effects. Integrated approaches involving comparative genomics, multi-omics, isotope tracing, and targeted genetic validation will be essential to establish causal relationships between sucrose metabolism and secondary metabolite biosynthesis. These approaches will facilitate identification of key metabolic bottlenecks limiting industrial productivity. Advances in synthetic biology and metabolic engineering offer additional opportunities for enhancing metabolite yields. Targeted manipulation of global regulators such as CcpA, CodY, ComA, and Spo0A may enable precise control of carbon flux and biosynthetic pathway activation. Similarly, engineering of NRPS and PKS systems could facilitate the generation of novel antimicrobial compounds with improved therapeutic properties [76]. Particular attention should be directed toward understanding the molecular mechanisms through which sucrose and molasses modulate the metabolic flux in bacteria. Elucidation of interactions such as QS, stress-response, and carbon catabolite regulation may reveal new strategies for maximizing antimicrobial metabolite production while maintaining fermentation efficiency. Furthermore, integration of inexpensive agro-industrial substrates with advanced bioprocess engineering approaches may significantly improve the economic viability of large-scale microbial metabolite production [113].

Sucrose and sucrose-rich substrates can support secondary-metabolite production in selected *Bacillus* strains, but the response depends on strain, substrate, medium, cultivation conditions, and metabolite type. Although carbon metabolism and regulatory pathways that directly link sucrose to specific regulatory mechanisms remain limited, highlighting the need for controlled and integrated studies to establish causality.

In conclusion, *B. subtilis* represents a highly versatile microbial platform for the production of valuable antimicrobial secondary metabolites. The growing understanding of sucrose-mediated metabolic regulation and molasses-supported fermentation systems provides new opportunities for developing sustainable, cost-effective, and industrially scalable processes for antimicrobial compound production. Future advances in systems biology, synthetic biology, and metabolic engineering are expected to further unlock the enormous biotechnological potential of this important microbial species.

## Figures and Tables

**Table 1:** Biosynthesis and regulation of secondary metabolites in *B. subtilis* and related *Bacillus* sp.

S.N.	Bacteria	Metabolite	Activity	Regulation	Reference
1	*B. mojavensis B. subtilis*, *B. velezensis*	Surfactin	Antibacterial, antifungal, anti-biofilm, biosurfactant	Spo0A, AbrB, CodY, DegU, and transition-state mediated production	[10,30,31,32]
2	*B. amyloliquefaciens*, *B. subtilis*, *B. velezensis*	Iturin A	Antifungal activity	Production is regulated by nutritional conditions	[6,33]
3	*B. mojavensis*, *B. subtilis*, *B. velezensis*,	Fengycin/Plipastatin	Antifungal activity	DegU/DegQ-dependent regulation	[22]
4	*B. amyloliquefaciens*, *B. subtilis*, *B. velezensis*,	Bacillomycin D/Bacillomycin L	Antifungal activity	Transition-state networks and environmental adaptation pathways	[34,35]
5	*B. subtilis* ATCC 6633	Mycosubtilin	Antifungal lipopeptide	Regulate growth phase, oxygen availability, and physiological stress	[36]
6	*B. licheniformis*	Lichenysin	Antibacterial, biosurfactant, anti-biofilm	Environmental stress and stationary phase affect biosurfactant production	[37]
7	*B. amyloliquefaciens*, *B. subtilis*, *B. velezensis*,	Bacilysin	Antibacterial and Antifungal activity	Positively regulated by Spo0A and Spo0H; negatively regulated by AbrB, CodY, and ScoC; affected by ppGpp/stringent response	[29,38]
8	*B. amyloliquefaciens*, *B. subtilis*, *B. velezensis*,	Bacillaene	Antibacterial	Spo0A, AbrB, Abh, CodY, DegU, and interspecies competition regulate *pks* expression	[39]
9	*B. amyloliquefaciens*, *B. velezensis*	Difficidin	Strong antibacterial, especially against plant-pathogenic bacteria	Regulatory complexes and environmental factor influence production	[40,41]
10	*B. amyloliquefaciens* FZB42	Macrolactin	Antibacterial; also reported antiviral and anti-biofilm activities	Regulation through *pks2* biosynthetic gene cluster	[42]
11	*B. subtilis*	Subtilosin A	Antibacterial, especially against Gram-positive bacteria	State specific regulation in response to temperature and pH affecting *sboA* expression	[43,44]
12	*B. subtilis* ATCC 6633	Subtilin	Antibacterial, mainly against Gram-positive bacteria	Production depends on growth phase and environmental conditions	[45]
13	*B. subtilis* 168	Sublancin	Antibacterial, especially against Gram-positive bacteria	Stress response, prophage-linked regulation, and ecological competition influence expression	[46]
14	*Bacillus* sp. Strain HIL Y-85,54728	Mersacidin	Strong antibacterial, including activity against Gram-positive pathogens	Production depends on growth phase and environmental physiology	[47]
15	*B. licheniformis*, *Bacillus* sp.	Bacitracin	Antibacterial, mainly Gram-positive bacteria	Production is linked to growth phase, cell-envelope stress, and resistance regulation	[48]

**Table 2:** Effects of different carbon sources on secondary metabolites production in *Bacillus* species.

S.N.	Metabolite	Strain	Carbon Source Used	Favoured Carbon Source	Mechanism Behind Enhanced Production	References
1	Surfactin	*B. subtilis* NCIB 3610	Sucrose, glucose, fructose, maltose, arabinose	Sucrose Escalated surfactin production by 4-fold compared to other carbon sources	Response associated with SacB/levan route rather than simple sucrose	[64]
2	Surfactin	*B. subtilis* YPS-32	Molasses, sucrose, galactose, fructose, starch glycerol and glucose	Molasses Supported to enhance Surfactin production.	srfAA-srfAD operon mediated	[65]
3	Biosurfactant	*B.subtilis* Al-Dhabi-130	Date molasses-based solid substrate, with optimized conditions	Molasses Escalated biosurfactant production.	Creating a carbon-rich condition favorable for extracellular amphiphilic metabolite secretion.	[66]
4	Lipopeptide	*B. subtilis* ANR 88	Molasses, banana peel, orange peel, whey, potato peel and bagasse	Molasses Lipopeptide with 0.24 g/L yield	NRPS/srfA dependent synthesis	[52]
5	Surfactin/biosurfactant	*B. subtilis* BS5	Glucose, sucrose, maltose, extract, molasses, lactose, galactose,	Molasses Surfactin production yield ~1.12 g/L	srfA operon-mediated synthesis	[53]
6	Surfactin, iturin, fengycin, lichenysin-like lipopeptides	*B. subtilis*	Molasses, sucrose, glucose, starch, glycerol and agro-industrial waste	Molasses Molasses expected to support production by supplying sugars and additional nutrients	Not mentioned	[67]
7	Iturin A	*Bacillus* sp. BH072	Glucose, sucrose, fructose, xylose, rhamnose and soluble starch	Sucrose Iturin A concentration reported as 52.21 mg/mL	Sucrose, along with soybean meal and Mg^2+^ enhanced production	[51]

**Table 3:** Genes involved in sucrose metabolism, carbon catabolite repression, quorum sensing and antimicrobial secondary metabolite regulation.

S.N.	Gene	Encoded Protein	Regulatory Response	Potential Relevance to Secondary Metabolite Production	References
1	*sacP*	PTS permease	Transports and phosphorylates sucrose	Facilitates sucrose uptake and carbon flow into central metabolism	[63,86]
2	*sacA*	Sucrose-6-phosphate hydrolase/intracellular sucrase	Induced by sucrose through sucrose-responsive regulatory pathways	Generates glucose-6-phosphate/fructose-derived intermediates	[87]
3	*sacT/sacY*	Transcriptional antiterminators for sucrose-responsive genes	*sacT* regulates *sacPA*; *sacY* regulates sucrose-dependent antitermination of *sacB*	Promotes efficient expression of sucrose-utilization genes,	[16,17,18,19,20,21,22,23,24,25,26,27,28,29,30,31,32,33,34,35,36,37,38,39,40,41,42,43,44,45,46,47,48,49,50,51,52,53,54,55,56,57,58,59,60,61,62,63,64,65,66,67,68,69,70,71,72,73,74,75,76,77,78,79,80,81,82,83,84,85,86,87]
4	*ccpA*	Catabolite control protein A; global CCR regulator	Mediates CCR in response to preferred carbon availability	May Affect secondary metabolism under carbon-rich conditions.	[88]
5	*ptsH*/HPr	PTS phosphocarrier protein	Phosphorylated at Ser46 under high glycolytic flux, HPr-Ser46-P- CcpA complex, strengthening CCR	Affects secondary metabolite synthesis through carbon-responsive regulation	[89]
6	*hprK*	HPr kinase/phosphorylase	Responds to ATP and fructose-1,6-bisphosphate generated during active carbon metabolism	Controls HPr-Ser46-P formation, required for CCR regulation	[90]
7	*comA*	Response regulator of quorum-sensing system	Activated indirectly when growth conditions permit high cell density and quorum signaling	ComA-dependent regulation affects surfactin biosynthesis through expression	[91]
8	*comP*	Membrane histidine kinase	Senses extracellular ComX pheromone at high cell density	Phosphorylates to ComA, leading to activation of *srfA* expression	[72]
9	*comX*	Quorum-sensing pheromone	Accumulates with increasing cell density during favorable growth	Activates ComP-ComA signaling and stimulates surfactin-associated secondary metabolism	[72]
10	*spo0A*	Master regulator of transition state and sporulation	Activated during nutrient transition/stationary-phase entry	Represses *abrB*, relieving repression of secondary metabolite biosynthesis gene and developmental adaptation	[45]
11	*abrB*	Transition-state repressor	Repressed by Spo0A~P during transition phase	Relief of *AbrB* leads to developmental adaptation	[92]
12	*degU*	Global response regulator/two-component regulator	Influenced by environmental and physiological signals; effect is strain- and phosphorylation-state dependent	Modulates surfactin, fengycin/plipastatin, biofilm formation, and antimicrobial production; not always purely positive	[93]
13	*codY*	Nutritional-status regulator sensing amino acid/GTP availability	Active under nutrient-rich conditions; reduced activity during nutrient limitation	Derepression of *srfA* and nutrient-responsive secondary metabolite regulation	[94]
14	*srfAA-srfAD*	Surfactin non-ribosomal peptide synthetase complex	Activated mainly by ComA~P and transition-state regulation; sucrose/molasses may support expression indirectly through biomass and precursor supply	Direct biosynthesis of surfactin, an antimicrobial and biosurfactant lipopeptide	[73]
15	*fenA-fenE*/*ppsABCDE*	Fengycin/plipastatin synthetase complex	May be enhanced under favorable carbon flux and strain-specific regulatory conditions	Produces fengycin/plipastatin-type antifungal lipopeptides	[95]
16	*bacABCDE* */bacABCDEF*+ *bacG/ywfG*	Bacilysin biosynthetic proteins	Induced mainly in transition/stationary phase; controlled by quorum-sensing and global regulators	Produces bacilysin dipeptide	[25]
17	*pksX/pksA-pksS*	Modular polyketide synthase enzymes	Influenced by growth phase, competition, and carbon-flux redistribution	Produces bacillaene in *B. subtilis*; and difficidin/macrolactin in *Bacillus* sp.	[39]
18	*bae*/bacillaene-associated genes	Bacillaene biosynthetic proteins	Activated during secondary metabolism and interspecies competition	Produces bacillaene	[6,39]

## Data Availability

The data produced in this research are available from the corresponding authors upon reasonable request.
